# Extremely Elevated High-density Lipoprotein Cholesterol and the Risk of Atrial Fibrillation: The Suita Study

**DOI:** 10.2188/jea.JE20240428

**Published:** 2025-09-05

**Authors:** Ahmed Arafa, Yuka Kato, Yoshihiro Kokubo

**Affiliations:** 1 Department of Preventive Cardiology, National Cerebral and Cardiovascular Center, Osaka, Japan; 2 Department of Public Health, Faculty of Medicine, Beni-Suef University, Beni-Suef, Egypt; 3 Division of Health Sciences, Osaka University Graduate School of Medicine, Osaka, Japan

**Dear Editor,**

High-density-lipoprotein cholesterol (HDL-C) is known to have cardioprotective effects. However, recent studies have shown that extremely elevated HDL-C levels are associated with an increased risk of cardiovascular disease (CVD) mortality,^1^ stroke,^2^ heart failure,^3^ cognitive impairment,^4^ and peripheral artery disease.^5^ Extremely elevated HDL-C levels may result from hyperalphalipoproteinemia, a condition potentially linked to risky variants of apolipoprotein C-III. These variants can cause HDL-C dysfunction and promote atherosclerosis.^6^ Additionally, certain genetic mutations have been shown to increase both dysfunctional HDL-C levels and CVD risks. These include deletions in the gene encoding endothelial lipase, loss-of-function mutations in the gene responsible for the transporter that allows HDL-C to deliver cholesterol back to the liver, and mutations in the gene encoding cholesterol ester transfer protein (CETP).^6^ In addition to genetic factors, alcohol consumption,^7^ thyroid dysfunction,^8^ and lipid-lowering drugs^9^ may also elevate HDL-C levels.

On the other hand, atrial fibrillation (AF) is a significant risk factor for CVD,^10^ and identifying individuals at increased risk for developing AF is essential for effective CVD prevention.^11^ However, the association between extremely elevated HDL-C levels and AF risk remains unclear. We, therefore, used data from the Suita Study, a population-based prospective cohort study investigating people residing in an urban city in Osaka, Japan, to explore this association.

The Suita Study population (two randomly selected cohorts including 7,814 participants recruited from 1989 to 1998 and a volunteer group including 546 participants recruited from 1992 to 2006), baseline assessment (questionnaire, clinical examination, blood and urine analysis, and electrocardiogram [ECG]), and biennial follow-up check-ups were described elsewhere.^12^ In the current study, we excluded those who did not attend the baseline assessment or any follow-up, had AF or atrial flutter at baseline, or lacked baseline data on HDL-C, leaving 6,872 participants for the analysis.

AF was diagnosed during the follow-up visits using a standard 12-lead resting ECG (Minnesota Codes [8-3-1 to 4]). Hospital records, including AF medication, history of catheter ablation treatment, routine check-ups, and death certificates, were thoroughly reviewed to confirm the diagnosis.^12^

HDL-C levels were measured at the baseline check-up. There is no universally accepted threshold for defining extremely elevated HDL-C levels. Previous studies have applied cut-offs ranging from 80 mg/dL^1^^,^^2^^,^^5^ to 100 mg/dL.^3^^,^^4^ When we plotted a spline curve to illustrate this relationship, the risk of AF began to rise when HDL-C levels approached 100 mg/dL (eFigure 1). As a result, a cut-off value of 100 mg/dL was applied to define extremely elevated HDL-C levels in this study. We also repeated the analyses at lower cut-off values.

SAS version 9.4 software (SAS Institute Inc., Cary, NC, USA) was used for analysis. The chi-squared test was used to detect differences in the risk factors assessed at baseline per incident AF and baseline HDL-C levels. Cox regression analysis was used to identify AF risk, measured using hazard ratios (HRs) and 95% confidence intervals (CIs), among those with HDL-C levels <40, 60–99, and ≥100 mg/dL compared to 40–59 mg/dL. Person-years of follow-up were calculated from the date of baseline examination to that of AF diagnosis, death, censoring, or the last health examination, whichever came first.

The results were adjusted for the following variables assessed at the baseline check-up: age (<50, 50–59, 60–69, and ≥70 years), sex, smoking (never, former, and current), alcohol consumption (never, former, light to moderate [<2 *go*/day] and heavy [≥2 *go*/day]; 1 *go* is equivalent to 23 g of ethanol), obesity (body mass index ≥25 kg/m^2^), hypertension (blood pressure ≥140/90 mm Hg or receiving medications), diabetes (fasting blood glucose ≥126 g/dL or receiving medication), low-density-lipoprotein cholesterol (LDL-C) (<130, 130–159, ≥160 mg/dL), and CVD (history of coronary heart disease or stroke).

Besides, we assessed the interaction of age, sex, and alcohol consumption with the association between HDL-C levels and AF risk. We also conducted sensitivity analyses by (a) changing the HDL-C reference group to 40–99 mg/dL, (b) changing the cut-off defining extremely elevated HDL-C levels to 90 and 95 mg/dL, (c) excluding those who reported fasting <8 hours before blood sampling (*n* = 241; 3.5%), (d) excluding participants who reported heavy alcohol consumption (*n* = 969; 14.1%), (e) excluding participants who reported receiving lipid-lowering drugs (*n* = 167; 2.4%), (f) excluding participants with leukocytosis (white blood cell count >10,000 cells/µL) (*n* = 75; 1.1%), and (g) and excluding participants with a history of CVD (*n* = 151; 2.2%).

The results showed that the mean HDL-C levels of the study population stood at 54.3 (standard deviation, 14.3) mg/dL. Older adults, men, current smokers, heavy alcohol consumers, obese, and those with hypertension, diabetes, or a positive history of CVD were more likely to develop AF (eTable 1). Compared to participants with HDL-C levels of 40–59 mg/dL, those with HDL-C levels ≥100 mg/dL had a higher prevalence of older age, heavy alcohol consumption, hypertension, and diabetes, yet a lower prevalence of men, current smoking, obesity, and a positive history of CVD (eTable 2).

During the 95,131 person-years of follow-up (median, 14.6 years), 308 participants developed AF. Participants with HDL-C levels ≥100 mg/dL, compared to HDL-C levels of 40–59 mg/dL, had a higher risk of developing AF, with HRs of 2.95 (95% CI, 1.10–7.97) in the model adjusted for age and sex and 2.92 (95% CI, 1.07–7.96) in the model further adjustment for lifestyle and clinical factors. The association was attenuated, yet remained statistically significant, after involving LDL-C in the model: HR 2.74 (95% CI, 1.00–7.50). Age, sex, and alcohol consumption showed no interaction with the associations between HDL-C levels and AF risk (*P*-values of interaction: 0.474, 0.512, and 0.249, respectively) (Table 1).

**Table 1.** Association between high-density lipoprotein cholesterol and atrial fibrillation risk

	High-density lipoprotein cholesterol levels			

	<40 mg/dL	40–59 mg/dL	60–99 mg/dL	≥100 mg/dL
Number of participants	1,020	3,601	2,224	27
Number of incident cases	55	178	71	4
Incidence/1,000 person-years	4.04	3.57	2.27	10.80
Model I	0.90 (0.66–1.22)	1 (Reference)	0.79 (0.60–1.04)	2.95 (1.10–7.97)
Model II	0.84 (0.61–1.14)	1 (Reference)	0.82 (0.62–1.09)	2.92 (1.07–7.96)
Model III	0.82 (0.60–1.23)	1 (Reference)	0.81 (0.61–1.07)	2.74 (1.00–7.50)

Model I: HRs (95% CIs) adjusted for age and sex.

Model II: model I + smoking, alcohol consumption, obesity, hypertension, diabetes, and cardiovascular disease.

Model III: model II + low-density lipoprotein cholesterol.

*P*-values for age, sex, and alcohol consumption interactions were 0.474, 0.512, and 0.249 respectively.

The conclusion did not materially change when HDL-C levels 40–99 mg/dL were assigned as a reference group or when we separately excluded participants who reported fasting <8 hours before blood sampling, participants receiving lipid-lowering drugs, participants with leukocytosis, and participants with a positive history of CVD. However, the association became statistically non-significant after heavy drinkers were excluded: HR 2.89 (95% CI, 0.91–9.18), most probably due to weakening statistical power because heavy drinkers represented more than 14% of the study population. In addition, applying lower cut-offs to define extremely elevated HDL-C levels led to significant attenuations in the association (eTable 3).

According to numerous guidelines, clinical concerns typically arise when HDL-C levels drop below 40 mg/dL, while levels above these thresholds are generally deemed desirable or optimal.^13^ However, our results, alongside previous findings,^1^^–^^5^ suggest that extremely elevated HDL-C levels may not be beneficial, challenging the notion that HDL-C is inherently “good cholesterol.” Extremely elevated HDL-C levels may increase AF risk due to the potential transformation of HDL into “dysfunctional HDL,” which promotes inflammation and oxidative stress,^6^ key contributors to atrial remodeling and arrhythmogenesis.^14^ Excessive HDL-C levels can impair endothelial function, leading to vascular stiffness and disrupted atrial electrophysiology.^6^ Genetic mutations, such as CETP deficiencies, linked to high HDL-C levels may further exacerbate AF risk by altering HDL functionality and lipid metabolism.^6^

On the other hand, our results suggested that, despite not reaching statistical significance, participants with HDL-C levels of 60–99 mg/dL were less likely to develop AF than those with HDL-C levels of 40–59 mg/dL (Table 1). The relationship between moderately elevated HDL-C levels and AF risk has yielded inconsistent findings in previous studies. However, a meta-analysis of 11 studies demonstrated a linear and negative correlation between HDL-C levels of 31–80 mg/dL and AF risk, with a summary relative risk of 0.97 (95% CI, 0.96–0.99) for a one-mmol/L increase in HDL-C levels.^15^ A more recent meta-analysis of 11 studies reported almost a similar conclusion, showing that a one-SD increase in HDL-C levels was associated with a 5% reduction in AF risk (HR 0.95; 95% CI, 0.90–1.01).^16^

The main strengths of this study included its focus on an understudied topic, the application of a prospective cohort design with a long follow-up period and biennial check-ups, the investigation of a randomly recruited sample stratified by 10-year age and sex from the general population, and the use of standardized methods to ascertain AF diagnosis. The primary limitation of this study was the small number of participants with HDL-C levels ≥100 mg/dL and incident AF cases within this subgroup. Consequently, we were unable to stratify our findings based on various AF risk factors. Nonetheless, interaction tests and sensitivity analyses indicated that these risk factors did not significantly influence the relationship between HDL-C levels and AF risk. Another limitation is the lack of data about some AF risk factors, such as sleep apnea and thyroid dysfunction.

In conclusion, extremely elevated HDL-C levels (≥100 mg/dL) may be associated with a higher risk of AF, suggesting that they should not be considered cardioprotective. Future studies, therefore, should classify HDL-C levels into low, optimal, and very high categories to more accurately assess and predict AF. Further studies with larger numbers of subjects are needed to confirm our findings.

## SUPPLEMENTARY MATERIAL

eTable 1. Characteristics of participants by their atrial fibrillation status

eTable 2. Characteristics of participants by their high-density lipoprotein cholesterol status

eTable 3. Sensitivity analyses of the association between high-density lipoprotein cholesterol and atrial fibrillation risk

eFigure 1. High-density-lipoprotein cholesterol and atrial fibrillation risk. Age- and sex-adjusted hazard ratios and their 95% confidence intervals were calculated per Model II, and Lipoprotein(a) levels were modeled using restricted cubic splines with knots at the 5^th^, 25^th^, 50^th^, 75^th^, and 95^th^ percentiles. A reference value of 53 mg/dL (median) was applied.
