# Supplementary material for: Extremely Elevated High-density Lipoprotein Cholesterol and the Risk of Atrial Fibrillation: The Suita Study
Source: J Epidemiol. 2025 Sep 5;35(9):420–2. doi: 10.2188/jea.JE20240428 (PMC12358252; doi:10.2188/jea.JE20240428)
Supplement: Supplementary file 1 [file je-35-420-s001.pdf]

eTable 1. Characteristics of participants by their atrial fibrillation status

Risk factors		No atrial fibrillation	Atrial fibrillation	P-value*
Number of participants		6564	308	--
Age, %	<50 years	33.5	10.1	<0.001
	50–59 years	24.4	18.8	
	60–69 years	25.2	41.9	
	≥70 years	16.9	29.2	
Men, %		46.1	65.9	<0.001
Current smoking, %		28.1	32.1	<0.001
Heavy alcohol consumption, %		13.8	20.5	0.002
Overweight or obesity, %		19.7	29.6	<0.001
Hypertension, %		31.9	48.7	<0.001
Diabetes, %		4.9	8.1	0.013
LDL-C ≥160 mg/dL, %		17.4	17.5	0.771
HDL-C ≥100 mg/dL, %		0.4	1.3	<0.001
History of cardiovascular disease, %		2.1	5.2	<0.001

HDL-C, high-density-lipoprotein cholesterol; LDL-C, low-density-lipoprotein cholesterol.

*chi-squared tests were used

eTable 2. Characteristics of participants by their high-density lipoprotein cholesterol status

Risk factors		High-density-lipoprotein cholesterol levels				P-value*
		<40 mg/dL	40–59 mg/dL	60–99 mg/dL	≥100 mg/dL	
Number of participants		1020	3601	2224	27	--
Age, %	<50 years	25.4	32.4	35.9	26.0	<0.001
	50–59 years	22.9	23.9	25.1	22.2	
	60–69 years	31.8	25.7	23.6	29.6	
	≥70 years	19.9	18.0	15.4	22.2	
Men, %		71.8	50.0	30.9	33.3	<0.001
Current smoking, %		45.2	29.6	18.4	14.8	<0.001
Heavy alcohol consumption, %		15.0	13.8	14.1	18.5	0.156
Overweight or obesity, %		34.2	21.8	11.3	0.0	<0.001
Hypertension, %		36.4	33.2	29.6	51.9	<0.001
Diabetes, %		7.7	5.3	3.6	7.4	<0.001
LDL-C ≥160 mg/dL, %		16.6	19.9	14.0	3.7	<0.001
History of cardiovascular disease, %		4.2	2.1	1.5	0.0	<0.001

LDL-C, low-density-lipoprotein cholesterol.

*chi-squared tests were used

eTable 3. Sensitivity analyses of the association between high-density lipoprotein cholesterol and atrial fibrillation risk

Changes	High-density-lipoprotein cholesterol levels			
	<40 mg/dL	40–59 mg/dL	60–99 mg/dL	≥100 mg/dL
Changing HDL-C reference group	1 (Reference)		0.85 (0.65–1.12)	3.02 (1.11–8.21)
Removing participants who fasted <8 hours	0.79 (0.57–1.10)	1 (Reference)	0.81 (0.60–1.08)	3.00 (1.10–8.20)
Removing heavy alcohol consumers	0.93 (0.66–1.31)	1 (Reference)	0.82 (0.59–1.13)	2.89 (0.91–9.18)
Removing lipid-lowering drug users	0.87 (0.63–1.19)	1 (Reference)	0.88 (0.66–1.16)	2.92 (1.07–7.96)
Removing participants with leukocytosis	0.82 (0.60–1.12)	1 (Reference)	0.81 (0.61–1.08)	2.89 (1.06–7.87)
Removing cardiovascular disease patients	0.86 (0.62–1.19)	1 (Reference)	0.87 (0.66–1.16)	3.05 (1.12–8.33)

HDL-C, high-density-lipoprotein cholesterol.

Hazard ratios (HRs) and their 95% confidence intervals (CIs) were adjusted for age, sex, smoking, alcohol consumption, obesity, hypertension, diabetes, and cardiovascular disease. When we changed the cut-off defining extremely elevated HDL-C levels to ≥90 mg/dL; HRs of atrial fibrillation risk for the HDL-C levels 60–89 and ≥90 mg/dL were 0.84 (95% CI, 0.63–1.11) and 1.16 (95% CI, 0.51–2.65), respectively. When we changed the cut-off defining extremely elevated HDL-C levels to ≥95 mg/dL; HRs of atrial fibrillation risk for the HDL-C levels 60–94 and ≥95 mg/dL were 0.81 (95% CI, 0.61–1.08) and 2.26 (95% CI, 0.99–5.16), respectively.

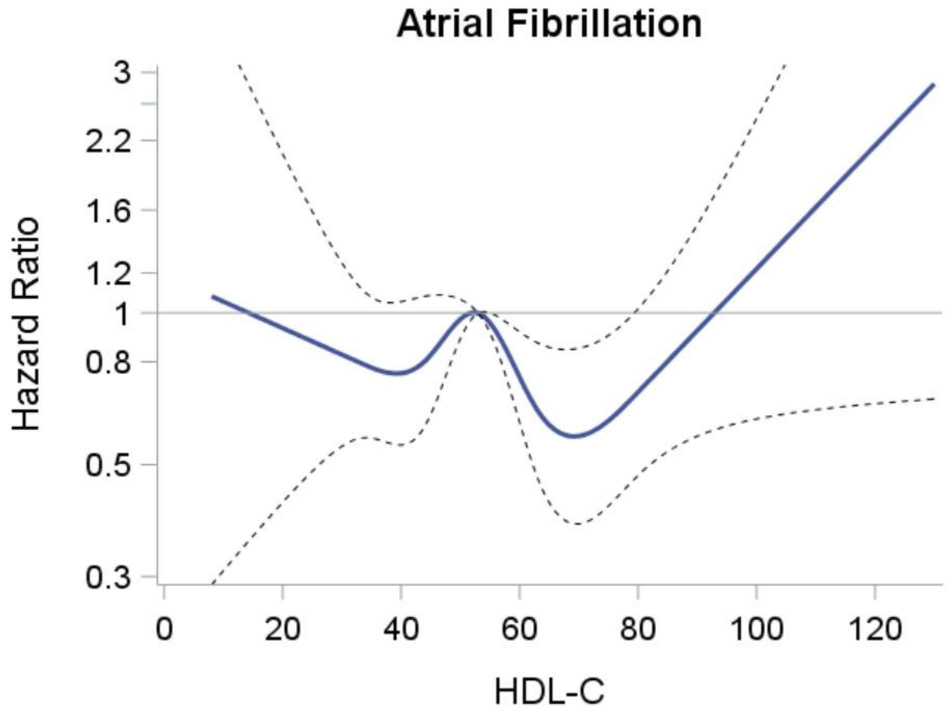

eFigure 1. High-density-lipoprotein cholesterol and atrial fibrillation risk. Age- and sex-adjusted hazard ratios and their 95% confidence intervals were calculated per Model II, and Lipoprotein(a) levels were modeled using restricted cubic splines with knots at the 5th, 25th, 50th, 75th, and 95th percentiles. A reference value of 53 mg/dL (median) was applied.
